# New Approach in Translational Medicine: Effects of Electrolyzed Reduced Water (ERW) on NF-κB/iNOS Pathway in U937 Cell Line under Altered Redox State

**DOI:** 10.3390/ijms17091461

**Published:** 2016-09-01

**Authors:** Sara Franceschelli, Daniela Maria Pia Gatta, Mirko Pesce, Alessio Ferrone, Antonia Patruno, Maria Anna de Lutiis, Alfredo Grilli, Mario Felaco, Fausto Croce, Lorenza Speranza

**Affiliations:** 1 Department of Medicine and Science of Aging, University of Gabriele D’Annunzio, 66100 Chieti, Italy; s.franceschelli@unich.it (S.F.); daniela.gatta@unich.it (D.M.P.G.); alessioferrone@yahoo.it (A.F.); antonia.patruno@unich.it (A.P.); maria.delutiis@unich.it (M.A.d.L.); mfelaco@unich.it (M.F.); 2 Medicine and Health Science School, University of Gabriele D’Annunzio, 66100 Chieti, Italy; mirkopesce@unich.it (M.P.); algrilli@unich.it (A.G.); 3 Department of Farmacy, University of Gabriele D’Annunzio, 66100 Chieti, Italy; fausto.croce@unich.it

**Keywords:** electrolyzed reduced water, antioxidant enzymes, nitric oxide, superoxide anion

## Abstract

It is known that increased levels of reactive oxygen species (ROS) and reactive nitrogen species (RNS) can exert harmful effects, altering the cellular redox state. Electrolyzed Reduced Water (ERW) produced near the cathode during water electrolysis exhibits high pH, high concentration of dissolved hydrogen and an extremely negative redox potential. Several findings indicate that ERW had the ability of a scavenger free radical, which results from hydrogen molecules with a high reducing ability and may participate in the redox regulation of cellular function. We investigated the effect of ERW on H_2_O_2_-induced U937 damage by evaluating the modulation of redox cellular state. Western blotting and spectrophotometrical analysis showed that ERW inhibited oxidative stress by restoring the antioxidant capacity of superoxide dismutase, catalase and glutathione peroxidase. Consequently, ERW restores the ability of the glutathione reductase to supply the cell of an important endogenous antioxidant, such as GSH, reversing the inhibitory effect of H_2_O_2_ on redox balance of U937 cells. Therefore, this means a reduction of cytotoxicity induced by peroxynitrite via a downregulation of the NF-κB/iNOS pathway and could be used as an antioxidant for preventive and therapeutic application. In conclusion, ERW can protect the cellular redox balance, reducing the risk of several diseases with altered cellular homeostasis such as inflammation.

## 1. Introduction

Oxidative stress is a phenomenon which results from the particular condition of balance between oxidative and reductive processes that continually occur in every cell in the complex biochemical transformations of the physiological metabolism [1]. The free radicals are important regulators of many physiological and pathological processes. The atoms are chemically stable when they are paired with each other; they are defined as free radicals when they are characterized by the presence of an unpaired electron [2]. Hence, the free radical is very unstable, extremely reactive and has a short half-life; in fact it reacts with nearby molecules [3]. These molecules become electrically unstable, triggering a series of chain reactions, which amplify the phenomenon and thus the number of free radicals produced, such as reactive oxygen species (ROS) and reactive nitrogen species (RNS) with production of superoxide (O_2_^−^), hydrogen peroxide (H_2_O_2_), nitric oxide (NO), and peroxynitrite (ONOO^−^) [4]. It has been demonstrated that ROS and RNS, at physiological low levels, act as signaling messengers to mediate several biological responses, such as proliferation, immunity, apoptosis, aging and gene expression [5,6]. Moreover, it is also known that elevated levels of these molecules can have toxic effects, altering the cellular redox state. In fact, documented evidence suggests that a disturbance in the ROS/RNS balance plays a pivotal role in several pathologic conditions. ROS and RNS are involved in various endogenous metabolic processes such as protein and DNA oxidation, energy generation, lipids peroxidation, nitration, nitrosation or nitrosylation and neurotransmitter response [7]. Generally, the initial formation of ROS and RNS radicals can be blocked by antioxidants’ endogenous enzymes or natural compounds. A number of reactive radicals are simultaneously produced by H_2_O_2_-stimulated cells, including O_2_^−^ and NO. NO is synthesized from l-arginine, by inducible NO synthase (iNOS) in macrophages and by proinflammatory stimuli. The amount of iNOS expression can be determined by the percentage of transcription, which is dependent on the translocation of NF-κB into the nuclei [8,9,10]. When the O_2_^−^ formation and NO synthesis were stimulated to a greater rate, NO was quantitatively converted to peroxynitrite. These last RNS may cause cellular toxicity by nitrative protein modification. The cells counteract the oxidative stress through antioxidant enzymes such as superoxide dismutase (SOD), catalase (CAT), glutathione peroxidase (GPx) and glutathione reductase (GR), which detoxify ROS, converting them into less reactive species [11].

Antioxidants are substances that can protect cells against the side effects of drugs, xenobiotics, carcinogens and toxic radical molecules. Several natural compounds have been reported to have antioxidant functions [1,2,3,4,5,6,7,8,9,10,11,12]. Recently, it was disclosed that Electrolyzed Reduced Water (ERW), generated at the cathode during water electrolysis, has high pH, low dissolved oxygen and an extremely negative redox potential (ORP). It has also been shown to exhibit scavenging activity [13]. In Taiwan, ERW is used as a source of drinking water and it has also gained much attention in Japanese research [14]. ERW can act as a scavenger of various free radicals such as H_2_O_2_, the hydroxyl radical (OH**·**), and the O_2_^−^ in cultured cells; protecting DNA, RNA proteins and cells against excessive oxidative stress. The defence mechanism of ERW is brought about by active atomic hydrogen, which has a high reducing power; it may also be involved in the redox regulation and can confer to scavenging activity [15]. Several studies have shown that natural antioxidants are able to prevent oxidative stress-related pathologies [16,17,18,19,20]. One of these antioxidants is ERW, which was selected in the present study. The study analyzed the antioxidant effect of ERW on H_2_O_2_-induced U937 damage by evaluating the modulation of redox cellular state. Here we demonstrated that cells cultured in an ERW-medium can alleviate H_2_O_2_-induced cytotoxicity and also proposed that ERW has potential as an antioxidant for preventive and therapeutic application.

## 2. Results

### 2.1. Chemical Characterization of ERW

Table 1 shows the value of redox parameters (*Eh*, *rH*_2_) of ERW with respect to tap water, MQ-water and MQ-water containing NaOH 2 mM. Marked changes in these values occur in ERW. The values were shown as Mean ± S.D. The *Eh* represents the redox potential of an aqueous solution and measured the reductive power ability of dissolved hydrogen. With respect to tap water, ERW has *Eh* and *pH* values that are rarely encountered in a natural environment. Concerning redox potential, values of ERW are strongly electronegative (−210.73 mV). The lower *Eh* value signifies the high reducibility of ERW due to the dissolved hydrogen gas produced by the electrochemical reaction in the cathode [21]. In fact, it can be seen that the main effect of electrolysis is a substantial reduction in the *rH*_2_ value of ERW with respect to tap water. After correcting—for the effect of *pH* and temperature—the *rH*_2_ (molecular hydrogen) was found to be 11.81, indicating that this medical device can efficiently produce water that is notably anti-oxidizing. The *Eh* and *rH*_2_ values revealed a reducing nature of the ERW that was stronger than tap water and MQ-NaOH. The ERW used has a high *pH* (9.47), high reducing power (*rH*_2_ = 11.81), and low *Eh* (−210.73 mV) compared with MQ-NaOH (*pH* = 9.5; *rH*_2_, 35.14; *Eh*, 477.60 mV).

### 2.2. ERW Protects Against H_2_O_2_-Induced Toxicity

**Figure 1:**
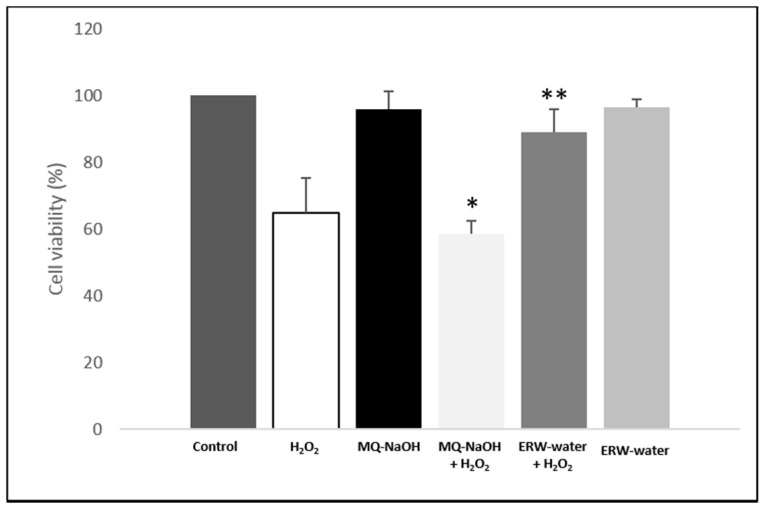
Cytotoxic effect ERW-medium on U937 cells line. Cells were grown in an MQ-medium or MQ-NaOH medium or ERW-medium with/without H_2_O_2_ and subjected to an MTT assay to analyse cell cytotoxicity. Data is presented as means ± SD for triplicate experiments. * *p* < 0.05 vs. MQ-NaOH; ** *p* < 0.05 vs. MQ-NaOH + H_2_O_2_.

ERW has been reported to suppress H_2_O_2_-induced oxidative stress in neuroblastoma N1E115 cells and adrenal gland PC12 cells [13,14,15,16,17,18,19]. We first examined whether H_2_O_2_-induced oxidative stress was suppressed by ERW in other cell lines, such as U937, a human leukemic monocyte lymphoma cell line. Different H_2_O_2_ concentrations at different time frames were tested in the present studies (data not shown). In our study, cells were exposed to 500 µM of H_2_O_2_ for 4 h with an MQ, MQ-NaOH or ERW-medium. As shown in Figure 1, compared to the control (MQ-NaOH); cell viability was decreased, after being treated with H_2_O_2_ (MQ-NaOH + H_2_O_2_) (*p* < 0.05), but ERW reversed this effect of H_2_O_2_. No significant changes were observed between cells grown in an ERW-medium or MQ-NaOH-medium, neutralized with bicarbonate buffer before use, with respect to cells grown in an MQ-medium (control). Therefore, the results indicate that ERW suppresses cell death caused by H_2_O_2_-induced oxidative damage.

### 2.3. ERW Suppressed H_2_O_2_-Induced Oxidative Stress Production

**Figure 2:**
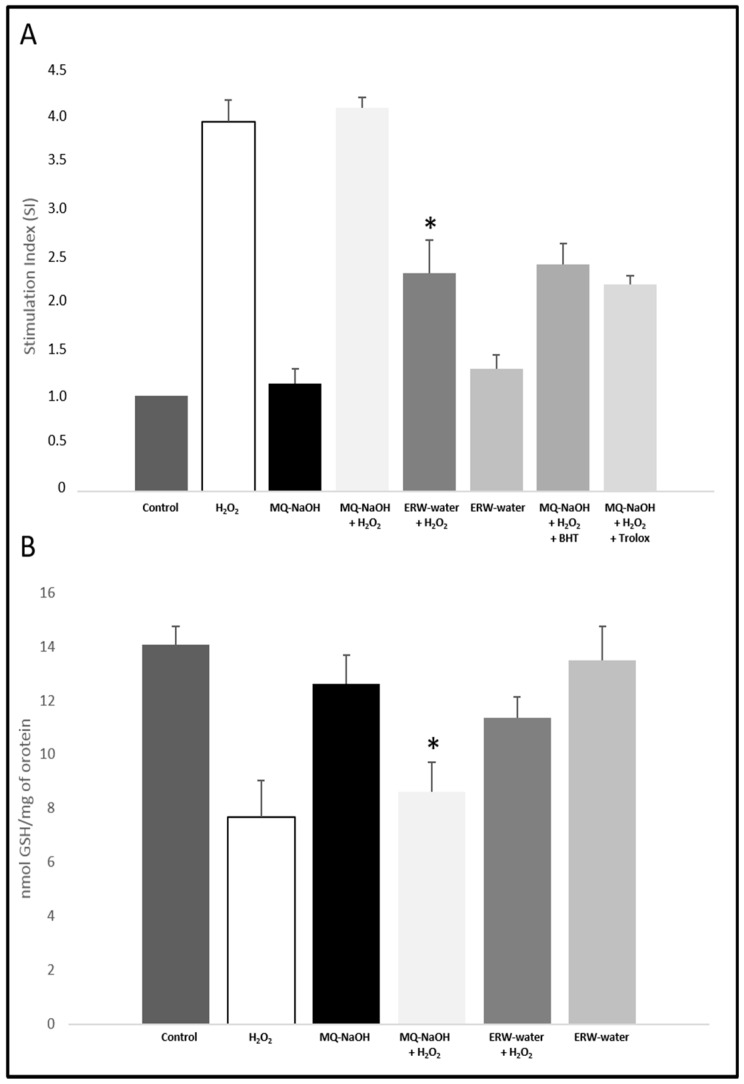
(**A**) Antioxidant activity of ERW water against oxidative stress measured by NBT test. Results were registered as stimulation index (SI). SI value of 1 was assigned to control cells; (**B**) effect of ERW water on GSH levels in U937 cells. * *p* < 0.05 vs. MQ-NaOH + H_2_O_2_. Data are presented as means ± SD for triplicate experiments.

To determine whether ERW might inhibit H_2_O_2_-induced ROS production, we performed the Nitro Blue Tetrazolium (NBT) reduction assay to measure ROS in U937 cells. The results were demonstrated through an NBT reduction stimulation index (SI), calculated as the optical density (OD) ratio of both control and treated cells. The SI for the control was taken to be one. Butyl hydroxy toluene (BHT) and Trolox were used as a positive scavenger control for ROS. As seen in Figure 2A, exposure to H_2_O_2_ resulted in increases in intracellular ROS production (4.11 ± 0.28) compared to the control (1.00). We observed the same trend when cells were cultured in a medium alkalinized with NaOH and buffered with bicarbonate. When the cells were grown in an ERW-medium, ROS production was reduced by 43% (*p* < 0.05) with respect to the cells grown in an MQ (NaOH)-medium + H_2_O_2_. The antioxidant effect of ERW is comparable to BHT scavenger activity. At the same time to confirm this data, GSH—one of the main defense systems against oxidative stress—was taken into consideration. Figure 2B demonstrated that H_2_O_2_ treatment decreased GSH content significantly whilst an ERW-medium increased it (*p* < 0.05).

### 2.4. Effect of ERW on Antioxidant Enzymes

**Figure 3:**
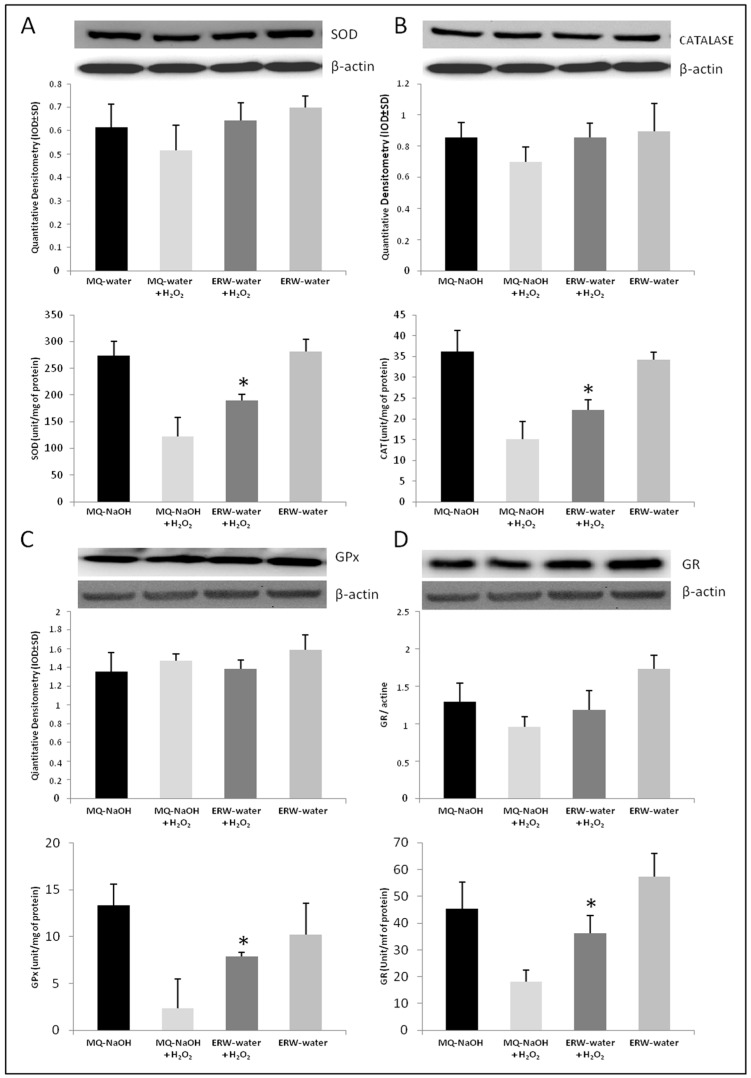
Effect of an ERW medium on the protein expression (**top**) and activity (**bottom**) of antioxidant enzyme SOD (**A**); CAT (**B**); GPx (**C**); and GR (**D**). * *p* < 0.05 vs. MQ-NaOH + H_2_O_2_. Data is presented as means ± SD for triplicate experiments.

Subsequently, protein expression of the antioxidant enzymes SOD, CAT, GPx and GR was tested in cultures of U937 cells grown in MQ-NaOH or an ERW medium with and without H_2_O_2_. The results referred to a constitutive control for protein expression, such as β-actin The results also showed no significant changes in the protein levels of SOD, CAT, GPx and GR with respect to control cells cultured in a medium suspended in MQ-NaOH, and in cells submitted to an ERW medium with respect to cells treated with MQ-NaOH + H_2_O_2_ (Figure 3A–D at the top). Instead, the activity of these antioxidative enzymes was modulated in cells grown in an ERW-medium compared to cells cultured in an MQ-NaOH medium and treated with a pro-oxidant stimulus. The presence of H_2_O_2_ in the culture medium induced significant redox deregulation, with a decrease in the enzyme activities of SOD, CAT GPx and GR (Figure 3A–D at the bottom). The H_2_O_2_ treatment decreases the antioxidant enzyme activity, however, when the cells were cultured in an ERW-medium the activity was restored. Taken together, these results suggest that an ERW-medium significantly decreases H_2_O_2_-induced oxidative stress in a U937 cell line.

### 2.5. ERW Influence NF-κB/iNOS Pathway and 3-Nitrotyrosine Protein

**Figure 4:**
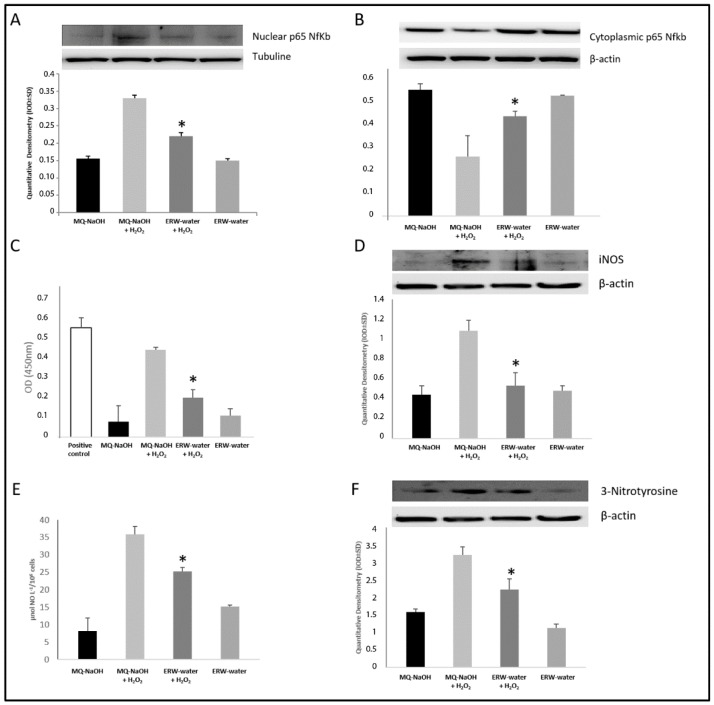
Effects of an ERW-medium on NF-κB, iNOS and 3-nitrotyrosine in U937 cells. Representative image of Western blot analysis for nuclear-p65 NF-κB (**A**); cytoplasmic-p65 NF-κB iNOS (**B**); ELISA for NF-κB p65 DNA binding activity (**C**); western blot analysis for iNOS (**D**); NO levels (**E**); and 3-nitrotyrosine protein expression (**F**). (**A**) Averaged band density of nuclear NF-κB from U937 cells normalized versus tubuline; (**B**) Averaged band density of cytoplasmic fraction of p65 NF-κB from U937 cells normalized versus β-actine; (**C**) effect of ERW on NF-κB p65 DNA binding activity; (**D**) effect of an ERW-medium on preventing H_2_O_2_-induced iNOS expression in U937 cells. Averaged band density of iNOS from U937 cells normalized versus β-actin. Cells were treated or not treated with H_2_O_2_ and/or an MQ-NaOH/ERW-medium; (**E**) effects of an ERW-medium after 4 h of incubation with H_2_O_2_ on NO production in U937 cells. NO levels were quantified by the accumulation of nitrite in the cell culture medium and are expressed as µmol NO/L^−1^/10^6^ cells; (**F**) Effect of ERW on 3-nitrotyrosine formation. Values are mean ± SD of different experiments performed in triplicate. * *p* < 0.05 versus MQ-NaOH + H_2_O_2_ treated cells.

The antioxidant regulation of NF-κB/iNOS activation and gene expression, to which this subject is signaling, has been reported frequently [5,6,7,8,9,10,11,12,13,14,15,16,17,18,19,20,21,22]. Western Blotting analysis showed that H_2_O_2_ increased the nuclear protein expression of NF-κB with statistical significance, and treatment with ERW clearly reduced the nuclear translocation with respect to an MQ-NaOH medium + H_2_O_2_ treated cells (Figure 4A). In parallel, our experiments have shown—in cells co-stimulated with H_2_O_2_ and ERW—an increased presence of p65 NF-κB in the cytoplasmic fraction, demonstrating the lesser nuclear translocation of the transcription factor with respect to cells activated with H_2_O_2_ (Figure 4B). To define the involvement of the NF-κB pathway in the ERW-mediated suppression of the H_2_O_2_-induced oxidative stress; activity of the NF-κB signaling pathway was evaluated by measuring the NF-κB p65 DNA binding activity in nuclear extracts. As shown in Figure 4C, the H_2_O_2_-induced activation of NF-κB was blunted by ERW.

Because iNOS activation is regulated at the transcriptional level by NF-κB, we have analyzed whether or not ERW played a role in the modulation of inducible nitric oxide synthase protein and activity (Figure 4D). As expected, iNOS expression was induced in cells cultured in an MQ-NaOH based medium and co-treated with H_2_O_2_ (*p* < 0.05); this effect was, instead, reversed when the cells were activated with H_2_O_2_, but cultured in an ERW-medium (*p* < 0.001 vs. MQ-NaOH medium). This data is supported by the quantization of the accumulated nitrite, used as an index for NO synthesis from these cells estimated in the culture medium (Figure 4E). The induction of iNOS, leading to high levels of NO, has a high affinity with O_2_^−^ with peroxynitrite and the 3-nitrotyrosine formation. This is highlighted in our experiments, as H_2_O_2_ induced an increase in the 3-nitrotyrosine formation in U937 cells, indicating the presence of nitrosative stress. This effect was smaller when cells activated with H_2_O_2_ were grown in ERW (Figure 4F).

## 3. Discussion

Oxidative stress is a particular condition induced by an exaggeration of the dynamic equilibrium between pro-oxidant oxidative and reductive processes that occurs continuously in each cell, with physiological expression of complex biochemical transformations of the terminal metabolism [23,24]. ERW, obtained by electrolysis, has been shown to exhibit H_2_O_2_-scavenging activity, which is correlated with; protection of DNA; alloxan-induced type 1 diabetes; carbon tetrachloride induced liver damage; hemodialysis-induced oxidative stress, during end-stage renal disease; and inhibitory effects of human fibrosarcoma tumor cell invasion [14,15,17,25,26,27,28]. Despite the various protective functions exhibited by ERW, its effect on U937 cells has not been disclosed in the literature; we have instead reported the role of ERW on H_2_O_2_-induced cultured U937 cells’ oxidative stress. ERW, produced near the cathode during electrolysis by an *Alkavitha* medical device, has characteristics of high *pH*, an extremely negative *Eh* and a high reducing power (*rH*_2_) (Table 1). The hydrogen molecules produced in the cathode represent a most important reducible chemical species dissolved in ERW. In order to investigate the effect of ERW on the alteration of redox balance, U937 cells were grown in an ERW-medium and exposed to 500 µM of H_2_O_2_. As shown in Table 1, a freshly prepared ERW-medium contained enough dissolved hydrogen molecules—rarely encountered in a natural environment. Moreover, the cells activated with H_2_O_2_ and cultured in an ERW-medium showed a recovery in cell viability compared to cells grown in MQ-NaOH (Figure 1). Huang et al. claim that the protective mechanism of ERW derives from molecular hydrogen with a high reducing ability, and that this could contribute to ROS scavenging activity, as well as participate in the cellular redox regulation [29]. Furthermore, Tsai et al. reported that ERW, produced by electrolysis, had no effect on the cell viability of PBMCs, isolated from healthy donors. They reported that ERW and GSH protect normal cells, however they inhibit the growth of cancer cells [14]. In our study, H_2_O_2_-treated U937 cells, grown in an ERW-containing medium, exhibited significantly lower intracellular O_2_^−^ levels than cells grown in an MQ-NaOH medium, showing that ERW probably scavenged intracellular O_2_^−^ (Figure 2A). A significant reduction in levels of O_2_^−^ was made evident, which plays an important physiological role at low concentration, such as signal transduction, apoptosis, cell proliferation and differentiation. Generally, in the cells, the anion O_2_^−^ is physiologically removed from the dismutation reaction by the enzyme SOD in H_2_O_2_, and consequently converted to water by catalase and glutathione peroxidase. When the cells are in a state of high stress—characterized by an excessive increase in O_2_^−^—catalytically active metals Fe^2+^ and Cu^+^ present react with H_2_O_2_, a strong oxidizer, producing high concentrations of highly reactive OH radicals via the Fenton or Weise reaction.

Our data is in accordance with in vitro research experiments by Shirahata and his group, which made evident that ERW neutralizes ROS, that is, a very similar process to the action of SOD and CAT enzymes [15]. SOD is one of the primary mitochondrial antioxidants in a network of detoxification enzymes. Therefore, if there is a reduction in the SOD, it is likely that there will be a further reduction in the catalytic activity of the subsequent detoxification enzymes.

The activities or expressions of antioxidant enzymes in cells are regulated by transcriptional, translational, and/or post-translational mechanisms, as a consequence of changes in the cellular redox potential. A potential mechanism for the reduction of antioxidant enzyme activities may be due to the inactivation caused by the excess of free radicals [30].

In our experiments, in a condition of H_2_O_2_-induced oxidative stress, we observed a reduced activity of SOD and catalase (Figure 3A,B), which causes the oxidation of Fe^2+^/Cu^+^ with O_2_^−^ reduction in the OH radical. This accumulation in our experimental set tends to decrease in activated monocytes cultured in an ERW-medium, where our evaluations evidenced a significant presence of the reducing agent H_2_ (Table 1). This is particularly important as restoring the redox state leads to the cell being in a state of homeostasis. Our results are further confirmed by the analysis of selenoprotein GPx, which represents an important antioxidant defense of the cells that are relevant for the protection against oxidative damage induced by H_2_O_2_. The activity of GPx markedly decreased in H_2_O_2_-treated cells due to the excessive concentration of the substrate, however it was significantly restored in ERW-cultured cells (Figure 3C). Moreover, consistent with these results, we also found that an ERW-medium can counter the oxidant attack and may protect cells against H_2_O_2_ damage, as reflected by the increase in GR activity and GSH levels (Figure 2B and Figure 3D). These results indicate that H_2_-induced glutathione homeostasis reestablishment exerts an important role in the alleviation of H_2_O_2_ stress through O_2_^−^ detoxification. Our results reflect the studies described above, which highlighted that ERW acts as a ROS scavenger due to the presence of hydrogen molecules that reestablish the antioxidant enzymes activity in SOD, catalase and GPx. Moreover, as high ROS levels have proven to assist in the activation and translocation of NF-κB into the nucleus, several inducible enzymes, such as iNOS, are overexpressed, contributing to an uncontrolled alteration of physiological homeostasis [31]. Therefore, in order to substantiate the antioxidant role of water, we examined the effects of ERW on H_2_O_2_-induced oxidative stress, on NF-κB/iNOS signaling. As expected, our results show that H_2_O_2_ activates NF-κB with enhanced iNOS expression (Figure 4A–D).

In agreement with our results already achieved, when the cells were grown in an ERW-medium, nuclear translocation of NF-κB was suppressed, and consequently iNOS expression is down-regulated. This led to a reduction of NO production through a mechanism that involves a restoration of antioxidant enzymes (Figure 4E). In a cell system that shows an alteration of the redox state, the NO released by this inducible enzyme rapidly reacts with superoxide anion, causing nitrosative stress and also generating highly oxidative peroxynitrite (ONOO^−^) [32]. This could lead to tissue damage by the nitration of aromatic amino acids such as tyrosine, yielding 3-nitrotyrosine; as well as a loss of protein structure and function [32,33,34]. Peroxynitrite is well known to inactivate SOD by nitrating a critical Tyr residue (Tyr 34) at the active enzyme site both in vitro and in vivo [35].

As shown in Figure 4F, in the cells activated, the NO reacts with O_2_^−^ yielding the oxidizing agent ONOO^−^, which leads to an increased production of 3-nitrotyrosine—a biomarker of ONOO^−^ formation—whereas ERW-cultured cells significantly attenuated the H_2_O_2_-induced increase of 3-nitrotyrosine expression. On the whole, our data suggested that ERW, with high *rH*_2_, acts as an O_2_^−^ scavenger, restoring the activity of antioxidant enzyme and the ability of the GR to supply the cell of an important endogenous antioxidant, such as GSH. Consequently, the negative effect of H_2_O_2_ on the redox balance of U937 cells is reversed and, in turn, this means a reduction in the cytotoxicity induced by ONOO^−^ via downregulation of the NF-κB/iNOS pathway. Therefore, molecular hydrogen can selectively reduce O_2_^−^ in vitro. The H_2_ dissolved in ERW has a number of benefits: Antioxidant potential; it effectively neutralizes the radical in living cells; it is able to penetrate through the biological membranes and can spread in the cytosol, mitochondria and nucleus [36,37,38]. Its ability to protect the cells suggests that it may reduce the risk of diseases related to alteration of homeostasis.

## 4. Materials and Methods

### 4.1. Apparatus Producing ERW and Procedure for the Measurement of ORP, pH, Eh and rH_2_

ERW was prepared by electrolysis from a municipal water system, using an electrolyzing medical device called *Alkavitha* (Vithagroup-Alkavitha, L’Aquila, Italy). The apparatus consists of two parts, one with an active carbon filter (0.2 µm) for water purification and the other with Pt-coated Ti electrodes for water electrolysis. Furthermore, the equipment for the electrolysis of water can control the pH regulator from *pH* 8.10 to 11.60 and *Eh* values from −200 to −800 mV.

*ORP* was measured using an ORP meter (Amel, Milano, Italy); *pH* was measured using a pH meter (Amel, Milano, Italy). All measurements were performed at a temperature of 25 °C. Unlike *ORP*; which yields a rough indicator of the amount of hydrogen reducing power available, but which is highly dependent of effects of *pH*; the relative hydrogen (*rH*_2_) yields a true index of hydrogen reducing power available, and fully compensated for *pH*, to allow removal of all of the H^+^ ion influence. For water values, 0 < *rH*_2_ < 28 has some reductive or antioxidant power, while values 28 < *rH*_2_ < 42 have an oxidation power [21]. The *rH*_2_ value was determined using the following equation:

$$rH_{2}=\frac{2}{S^{*}}\;Eh+2\;pH$$

where the factor is 33.8 at 25 °C and 33.4 at 20 °C and the *Eh* is the *ORP* value (expressed in volts) measured using a Pt as working and Ag/AgCl (in 3.33 mol/L KCl) as the reference electrode.

### 4.2. Preparation of Medium and Cell Culture

In order to investigate the effects of ERW on H_2_O_2_-induced oxidative stress in U937 cells (American Type Culture Collection, Manassas, VA, USA), an RPMI medium was prepared using MQ-water containing NaOH 2 mM (MQ-NaOH) or ERW supplied by the device. The ERW used has a high *pH* (9.4 ± 0.15), high reducing power (*rH*_2_ 11.81 ± 0.58), low redox potential (*ORP*) (−210.73 ± 24.51 mV) compared with MQ-NaOH (*pH*, 9.5 ± 0.08; *rH*_2_, 35.14; *ORP*, 477.6 ± 2.82 mV). The values were shown as Mean ± S.D. The ERW-medium and MQ-NaOH-medium were neutralized with bicarbonate buffer before use. The *pH*, *ORP* and *rH*_2_ of MQ-NaOH and ERW-based media were as follows: MQ-NaOH based medium (*pH*, 7.40 ± 0.08; *rH*_2_ 30.01 ± 0.18; *ORP*, 450.1 ± 7.77 mV) and ERW-based medium (*pH*, 7.48 ± 0.03; *rH*_2_, 22.91 ± 0.1; *ORP*, 240.1 ± 4.94 mV). A closed capped glass bottle was filled with freshly prepared ERW and stored in an inverted position to avoid loss of the dissolved hydrogen before preparation of the ERW-containing medium. RPMI 10× mediums were diluted by neutralized ERW or MQ-water as the control to make the control medium. The medium was immediately sterilized by filtration through a 0.2 µm filter under applied pressure. The U937 cells—a human leukemic monocyte lymphoma cell line—were cultured in MQ-NaOH or ERW containing a medium supplemented with 10% fetal bovine serum (FBS; Funakoshi, Tokyo, Japan). Due to the instability of the ERW over time (Table 1), in order to conserve the redox potential or *rH*_2_, cells were seeded (at 2 × 10^5^ cells per well) onto six-well tissue culture plates and cultured in a medium with H_2_O_2_ (500 µM) for 4 h, with an ERW or MQ-NaOH medium. Control cells were grown in a medium suspended in MQ-NaOH. The concentrations of H_2_O_2_ were chosen according to our preliminary optimization studies (data not show). After incubation, cells were harvested by centrifugation to assess cellular viability, protein expression and activity. Media of U937 cells was collected in order to evaluate the NO release. For the activities analysis, the harvested cells were suspended in a 10 mM phosphate buffer (pH 7.5) and then lysed on ice by sonicating twice for 15 s. Triton X-100 (1%) was then added to the lysates and incubated for 10 min on ice. The lysates were clarified by centrifugation at 5000× *g* for 10 min at 4 °C. The protein content of the supernatant was determined using the Bradford method.

### 4.3. MTT Assay for Cell Viability and Cytotoxicity

The MTT assay was used to assess cell damage by the oxidants and cell viability protection by the extracts [22]. Briefly, the U937 cells were seeded on 96-well plates at a density of 8 × 10^3^ cells/well, and cultured and treated according to the method described above. A total of 20 µL of MTT was added at a concentration of 0.5 mg/mL; after, a medium (200 µL) was added to each well. The plates were incubated at 37 °C for 4 h to dissolve the formazan that had formed. The solution (220 µL) was removed from each well and 150 µL of DMSO was added. The reduced MTT was measured on an ELISA reader (Bio-Rad, Hercules, CA, USA) at a wavelength of 570 nm. The cell viability percentage was calculated according to the equation below:

$$\%=\frac{\mathrm{Absorbance}\;\mathrm{of}\;\mathrm{treated}\;\mathrm{cells}}{\mathrm{Absorbance}\;\mathrm{of}\;\mathrm{control}\;\mathrm{cells}}\times 100$$

### 4.4. NitroBlue-Tetrazolium (NBT) Assay

This assay is used to detect the superoxide dismutation potential of the ERW. As described previously, cells were seeded on to 96-well culture plates at concentrations of 5 × 10^5^ mL^−1^ [39]. The following were added to each well: 100 μL potassium phosphate buffer, pH 7.8 (50 mM), 5 μL catalase, 25 μL NBT (5.6 × 10^−9^ M), 50 μL xanthine (0.1 mM), 50 μL xanthine oxidase (0.1 mM). Following the addition of NBT, the plates were allowed to stand at room temperature for 1 h until the blue color had developed and the absorbance was measured at 560 nm.

### 4.5. Western Blot Analysis

Total protein extracts were prepared by treating cells with the lysis buffer (RIPA). Nuclear extracts were prepared as previously described [40]. For nuclear extracts, cells were pelleted, frozen in dry ice/ethanol, resuspended in 75 μL of Buffer A (10 mmol·L^−1^ HEPES pH 7.9, 10 mmol·L^−1^ KCl, 0.5 mmol·L^−1^ EDTA, 1.5 mmol·L^−1^ MgCl_2_, 0.2% NP-40 and 0.5 mmol·L^−1^ PMSF) and placed on ice for 10 min to allow lysis. Nuclei were pelleted by centrifugation at 3500× *g* for 10 min at 4 °C, resuspended in 1 mL of Buffer B (20 mmol·L^−1^ HEPES pH 7.9, 400 mmol·L^−1^ NaCl, 1.5 mmol·L^−1^ MgCl_2_, 0.5 mmol·L^−1^ EDTA, 25% glycerol and 0.5 mmol·L^−1^ PMSF) and placed on a rocking platform for 30 min at 4 °C. The nuclear lysates were then clarified by centrifugation at 14,000× *g* for 20 min at 4 °C and the supernatants (nuclear extracts) collected.

Proteins were quantified using the Bradford Method. The Western Blot analysis was performed using 40 µg of protein and the following primary antibodies: SOD, catalase, Glutathione peroxidase, glutathione reductase, p65 NF-κB, iNOS and 3-nitrotyrosine (Santa Cruz Biotecnology, Santa Cruz, CA, USA) diluition 1:500). Protein levels were normalized to the housekeeping protein actin or tubuline to adjust for the variability in protein loading, and expressed as a percentage of the vehicle control.

### 4.6. Cu, Zn-Superoxide Dismutase (SOD) and Catalase Activity

The SOD assay mixture contained 50 mM sodium carbonate/ bicarbonate buffer (pH 9.8), 0.1 mM EDTA, 0.6 mM epinephrine and enzyme and 10 µg protein of enzymatic extract. Once epinephrine is added, adrenochrome formation was then read for 4 min at 475 nm in a UV-Vis spectrophotometer. Per unit, SOD activity is expressed as the amount of enzyme required for 50% inhibition of epinephrine oxidation. The CAT activity was measured spectrophotometrically. The decomposition of H_2_O_2_ was monitored continuously at 240 nm. The assay mixture, in a final volume of 3 mL, contained 10 mM of the potassium phosphate buffer, 10 mM of H_2_O_2_ and 5 μg of enzymatic extract protein. The CAT units were defined as 1 mole of H_2_O_2_ decomposed/min at 25 °C.

### 4.7. Glutathione Peroxidase (GPx) and Glutathione Reductase (GR) Activity

The quantification of the GPx activity was evaluated as previously described [7]. Briefly, the activity of Se-dependent GSH peroxidase was measured, with H_2_O_2_ (0.25 mM) as the substrate. The oxidation of NADPH was observed at 25 °C on a Hewlett and Packard spectrophotometer at 340 nm. One unit was defined as 1 mmol of GSH oxidized/min. The GR activity was spectrophotometrically monitored at 340 nm and 25 °C. The assay mixture in a final volume of 3 mL contained 0.1 mM of the potassium phosphate buffer, pH 7.4, 1 mM of EDTA, 1 mM of GSSG (Sigma-Aldrich, Milano, Italy), 0.16 mM of NADPH (Sigma) and 1–30 micrograms of U937 protein. One unit of enzyme activity was defined as 1 mmol of NADPH oxidized/min at 25 °C.

### 4.8. Measurement of GSH

The reduced glutathione (GSH) content of cells was determined by using the method described by Jollow et al. [41]. U937 cells cultured in 6-well plates were subjected to the indicated treatments, and lysed on ice for 30 min by RIPA lysis reagent. Cell lysate was clarified by centrifugation (10,000× *g*, 10 min, 4 °C), and the supernatant was collected for further detection. T-GSH was assayed using the 5,5-dithio-bis (2-nitrobenzoic) acid (DTNB)-GSSG reductase recycling. GSSG was measured by measuring 5-thio-2-nitrobenzoic acid (TNB) produced from the reaction of reduced GSH with DTNB. The rate of TNB formation was measured at 412 nm by the auto-microplate reader. The concentration of reduced GSH in the sample was obtained by subtracting GSSG from T-GSH. The color was read at 412 nm. GSH values are expressed as nmol GSH/mg proteins.

### 4.9. Measurement of NO Release

Briefly, 2 × 10^6^ cells were seeded in a 6-well/plate and nitrite was measured in culture supernatants as an indicator of the NO production. The assay was carried out as described previously [42]. Aliquots of the culture supernatant were mixed with an equal volume of the Griess reagent and absorbance was determined at 540 nm using a microplate reader. Sodium Nitrite, at concentrations of 0 to 100 μM, was used as a standard to assess nitrite concentrations.

### 4.10. Measurement of NF-κB p65 DNA Binding Activity

NF-κB p65 DNA binding activity in nuclear extracts was analyzed by a p65 NF-κB Transcription Factor Assay Kit (Abcam, Cambridge, UK), in accordance with the manufacturer’s instructions. The absorbance was determined using a microplate reader set at 405 nm. The specific double stranded DNA (dsDNA) sequence, containing the NF-κB response element, is immobilized onto the bottom of wells of a 96-well plate. NF-κB, contained in a nuclear extract, binds specifically to the NF-κB response element. p65 NF-κB is detected by addition of a specific primary antibody directed against p65 NF-κB. A secondary antibody, conjugated to HRP, is added to provide a sensitive colorimetric readout at 450 nm. For each experiment, triplicate samples were measured for statistical significance.

### 4.11. Statistical Analysis

The results were reported separately for: the control cells; the cells stimulated with H_2_O_2_; the cells grown in an MQ (NaOH)-medium; the cells grown in MQ (NaOH) and stimulated with H_2_O_2;_ the cells grown in an ERW medium w/o H_2_O_2_. All qualitative variables were summarized as frequency and percentage and all quantitative variables as mean and standard deviation (SD). An ANOVA test was applied to assess the comparison of the quantitative variables between the groups. In all statistical tests, the threshold of statistical significance is assumed to be equal to *p* < 0.05.

## 5. Conclusions

In summary, the H_2_ dissolved in ERW acts as an antioxidant, neutralizing the free radical. This is very important if we consider that oxidative stress contributes to the development of many inflammatory diseases. To this end, further investigations on the benefits of ERW on several diseases with altered cellular redox balance are needed.

## Figures and Tables

**Table 1:** Physicochemical parameters of Tap water, MQ water, ERW and MQ-water containing NaOH 2 mM. Values are expressed as mean ± SD.

	*Eh* (mV)	*rH*_2_	*pH*	T (°C)
Tap Water	531.1 ± 17.67	33.34 ± 0.43	7.54	20
MQ-water	511.1 ± 34.64	30.18 ± 0.84	6.3	20
MQ-NaOH	477.6 ± 2.82	35.14 ± 0.06	9.5	20
ERW	−210.73 ± 24.51	11.81 ± 0.58	9.47	20
ERW-medium	240.1 ± 4.94	22.91 ± 0.11	7.4	20
MQ-NaOH medium	450.1 ± 7.77	30.01 ± 0.18	7.4	20
